# Poorly differentiated large-cell neuroendocrine carcinoma of the paranasal sinus

**DOI:** 10.1590/0100-3984.2016.0184

**Published:** 2018

**Authors:** Helder Groenwold Campos, Albina Messias Altemani, João Altemani, Davi Ferreira Soares, Fabiano Reis

**Affiliations:** 1 Universidade Estadual de Campinas (Unicamp), Campinas, SP, Brazil

**Figure 1:**
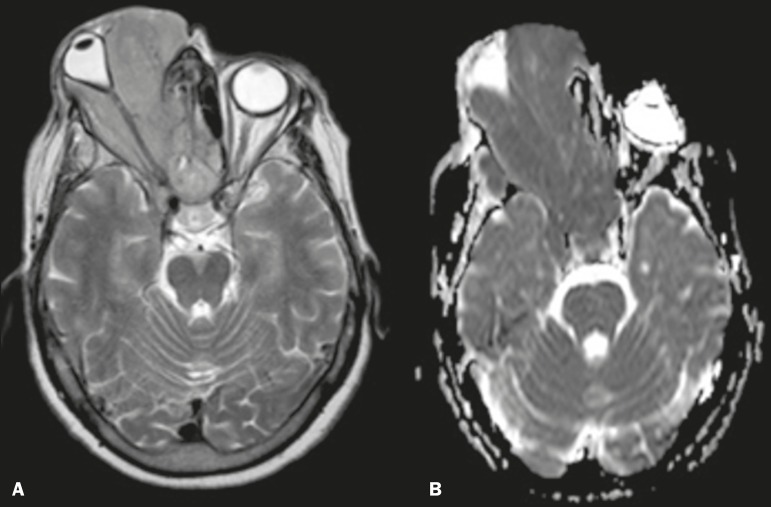
Mass occupying the right orbit and ethmoid sinus cells, laterally displacing the globe with isointensity on axial T2WI (**A**) and hypointensity onapparent diffusion coefficient mapping (**B**).

**Figure 2:**
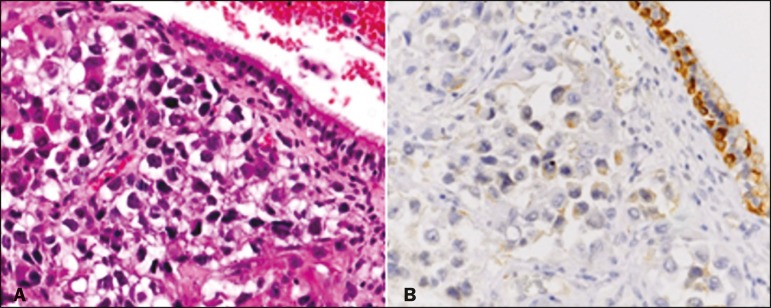
Immunohistochemical analysis demonstrating positivity for chromogranin A (**A**) and neural cell adhesion molecule or CD56 (**B**).

Dear Editor,

A 68-year-old female with a two-month history of rightsided ocular pruritus, progressive local edema, and wasting was referred for evaluation and biopsy of a soft-tissue mass, identified on a previous computed tomography (CT) scan, centered in the right orbit and extending to the maxillary, ethmoid, and sphenoid sinuses. At one month after the initial CT scan, she presented to our facility with proptosis, severe ocular pain, and ocular secretion. A new CT scan showed that the mass had expanded, invading the inferior and medial orbital walls, as well as the ethmoid bone, occupying the entire orbit and extending to the skin. On magnetic resonance imaging (MRI), the mass showed hypointensity on a T1-weighted image (WI) and isointensity on a T2WI, together with restricted diffusion, intense contrast enhancement, and skull base invasion (Figure 1). Immunohistochemical analysis demonstrated large poorly differentiated cells, numerous mitotic figures (> 10/high-power field), and foci of necrosis, as well as positivity for chromogranin A, CD56, and membrane epithelial antigen (Figure 2), rendering a diagnosis of large-cell neuroendocrine carcinoma (LCNC). Due its advanced stage (T4), the lesion was considered unresectable. The patient was started on radiotherapy and chemotherapy (cisplatin and etoposide) and responded well. At this writing, despite experiencing some adverse effects of the treatment (toxicity and optic nerve neuritis), she has been disease-free for 36 months.

Studies regarding head and neck tumors and pseudotumors are scarce in the recent radiology literature of Brazil^(1-5)^. Neuroendocrine carcinomas are a heterogeneous group of neoplasms that are most common in the lungs but are also found in the gastrointestinal tract and pancreas. The classification of neuroendocrine carcinomas remains controversial and usually follows the WHO 2004 Classification of Tumors of the Lung: well-differentiated neuroendocrine carcinoma (typical and atypical carcinoid); and poorly differentiated neuroendocrine carcinoma (small-cell neuroendocrine carcinoma and LCNC) ^(6-8)^. The diagnosis of LCNC is based on the following histologic and immunohistochemical criteria^(9)^: a high number of mitotic figures (> 10/high-power field); a low nuclear/cytoplasmic ratio; necrosis; and immunohistochemical positivity for at least one neuroendocrine marker (chromogranin A, neural cell adhesion molecule, or synaptophysin).

LCNC in the paranasal sinus is a rare presentation. The first case in the sinonasal region was described in 1982. Although the anatomical characteristics of the sinonasal region predispose to nonspecific clinical features initially, rapid growth can alter the presentation dramatically, with mass-effect related symptoms, as in the case presented here^(9)^. Imaging studies are essential for diagnostic and staging. On CT, a neuroendocrine carcinoma usually presents as a heterogeneous soft-tissue mass without calcifications and with strong contrast enhancement^(10,11)^. In one case series of patients with primary neuroendocrine carcinoma^(11)^, MRI showed hypointensity on T1WI in 91% of the cases and hyperintensity on T2WI in 83%, with intense contrast enhancement in all cases. Our case differs only in terms of the T2WI isointensity observed, which we believe reflects the high cellularity and low free-water content of the tumor. These characteristics are nonspecific, and it is not possible to differentiate neuroendocrine carcinoma from other more common etiologies, such as squamous cell carcinoma and lymphoma, on the basis of imaging findings alone^(12)^.

Staging follows the tumor-node-metastasis criteria, CT and MRI being complementary, due the better soft-tissue resolution of the latter, which allows better evaluation of skull base invasion. The evaluation of metastases should not rely on functional studies alone, because LCNC metastasis may lack octreotide/ somatostatin uptake^(13)^. Zhou et al. found that 81% of neuroendocrine carcinomas were at least stage T3 on presentation^(11)^. The rapid progression and advanced stage of the tumor at diagnosis denotes the malignant behavior of LCNC, which limits the proportion of patients who are candidates for surgery and, consequently, reduces survival.
